# Histological diagnosis of unprocessed breast core-needle biopsy via stimulated Raman scattering microscopy and multi-instance learning

**DOI:** 10.7150/thno.81784

**Published:** 2023-02-21

**Authors:** Yifan Yang, Zhijie Liu, Jing Huang, Xiangjie Sun, Jianpeng Ao, Bin Zheng, Wanyuan Chen, Zhiming Shao, Hao Hu, Yinlong Yang, Minbiao Ji

**Affiliations:** 1 State Key Laboratory of Surface Physics and Department of Physics, Human Phenome Institute, Academy for Engineering and Technology, Key Laboratory of Micro and Nano Photonic Structures (Ministry of Education), Yiwu Research Institute, Fudan University, Shanghai 200433, China; 2 Department of Breast Surgery, Fudan University Shanghai Cancer Center, Department of Oncology, Shanghai Medical College, Fudan University, Shanghai 200032, China; 3 Department of Pathology, Fudan University Shanghai Cancer Center, Shanghai 200032, China; 4 Endoscopy Center and Endoscopy Research Institute, Zhongshan Hospital, Fudan University, Shanghai, 200032 China; 5 Otolaryngology & Head and Neck Center, Cancer Center, Department of Otolaryngology, Zhejiang Provincial People's Hospital, Affiliated People's Hospital, Hangzhou Medical College, Hangzhou, Zhejiang, China; 6 Cancer Center, Department of Pathology, Zhejiang Provincial People's Hospital, Affiliated People's Hospital, Hangzhou Medical College, Hangzhou, Zhejiang, China

**Keywords:** breast cancer, core needle biopsy, label-free histology, stimulated Raman scattering, deep-learning

## Abstract

Core-needle biopsy (CNB) plays a vital role in the initial diagnosis of breast cancer. However, the complex tissue processing and global shortage of pathologists have hindered traditional histopathology from timely diagnosis on fresh biopsies. In this work, we developed a full digital platform by integrating label-free stimulated Raman scattering (SRS) microscopy with weakly-supervised learning for rapid and automated cancer diagnosis on un-labelled breast CNB.

**Methods:** We first compared the results of SRS imaging with standard hematoxylin and eosin (H&E) staining on adjacent frozen tissue sections. Then fresh unprocessed biopsy tissues were imaged by SRS to reveal diagnostic histoarchitectures. Next, weakly-supervised learning, i.e., the multi-instance learning (MIL) model was conducted to evaluate the ability to differentiate between benign and malignant cases, and compared with the performance of supervised learning model. Finally, gradient-weighted class activation mapping (Grad-CAM) and semantic segmentation were performed to spatially resolve benign/malignant areas with high efficiency.

**Results:** We verified the ability of SRS in revealing essential histological hallmarks of breast cancer in both thin frozen sections and fresh unprocessed biopsy, generating histoarchitectures well correlated with H&E staining. Moreover, we demonstrated that weakly-supervised MIL model could achieve superior classification performance to supervised learnings, reaching diagnostic accuracy of 95% on 61 biopsy specimens. Furthermore, Grad-CAM allowed the trained MIL model to visualize the histological heterogeneity within the CNB.

**Conclusion:** Our results indicate that MIL-assisted SRS microscopy provides rapid and accurate diagnosis on histologically heterogeneous breast CNB, and could potentially help the subsequent management of patients.

## Introduction

Breast cancer has become the most prevalent type of cancer and the leading cause of cancer-related death among women globally, accounting for ~25% of cancer cases and ~15% of cancer death in female patients 1, 2. Accurate diagnosis is critical for the treatment and prognosis of breast cancer 3. Percutaneous large core-needle biopsy (CNB) is a widely used practical approach for the initial diagnosis of breast cancer, especially for the clinically occult and nonpalpable breast lesions. CNB usually obtains tissue samples from suspected areas through a hollow core needle under the guidance of stereotactic mammography or ultrasound, which has been a commonly accepted alternative to open surgical biopsy with reduced trauma 4. Timely and precise histopathological evaluation of breast biopsy is important for the subsequent management of patients, yet it remains technically challenging.

Conventionally, H&E staining on biopsy tissues is required to provide the gold-standard histopathological information, including the cellular and nuclei morphology and extracellular tissue patterning 5. However, the series of time-consuming tissue preparation process is incompetent for rapid diagnosis on freshly excised CNB to provide timely medical guidance 6. In addition, the workflow of human-based diagnosis not only suffers from the shortage of professional pathologists, but also introduces inter-pathologist variation in grading different tumor subtypes 7-9. To reduce the burden of pathologists and obtain more objective diagnostic results, various machine-learning based classifiers have been developed for digital histopathology and automated disease grading 10-13. Therefore, an ideal platform for breast biopsy histopathology would expect the combination of rapid imaging technique with minimum tissue processing and automated diagnosis algorithm with high efficiency and accuracy.

Raman scattering has been well-known as a powerful spectroscopic tool for the detection and analysis of biological specimens and disease tissues, including breast cancer diagnosis 14-17. However, while conventional spontaneous Raman scattering is superior in spectral analysis, its intrinsic weak scattering efficiency prevents the biomedical applications in rapid imaging. Stimulated Raman scattering (SRS) overcomes such limitation by coherent excitation of molecular bond vibrations with preserved spectral fingerprints, enabling high speed, high chemical specific and three-dimensional imaging for various research fields 18-21. For label-free histology, SRS microscopy is able to provide histological information similar to traditional H&E, while bypassing the complex tissue sectioning or staining processes 22-28. It has demonstrated remarkable potentials in rapid histology for various types of human tissues and diseases, including brain tumor, laryngeal carcinoma, gastrointestinal tumor, pancreatic tumor and neurodegenerative disease, etc. 25, 29-35. The acquired chemical species commonly include lipid and protein imaged by SRS, and collagen fibers taken by second harmonic generation (SHG) 31, 36. On the other hand, various machine-learning models have been applied on SRS images for the analysis, classification and segmentation of tumor histopathology 29, 30, 37-39. Notably, Orringer et al. reported computer-aided diagnosis of brain tumors based on feature extractions 29, and convolutional neural networks (CNN) under supervised learning with homogeneously labelled histological classes as training datasets 30, 38, 39. And the classifications mostly rely on the area percentage of the predicted subgroup with a threshold (close to 50%) to reach a diagnostic result of the whole tissue slide. While this method works fine for relatively homogenous tissues such as brain, it may no longer be valid for breast biopsy because of the large tissue heterogeneity with significant amount of non-diagnostic tissues such as collagen fibers and adipocytes, which could vary drastically among specimens.

In contrast to the above statistics-based classification in supervised learnings, the diagnostic criteria of a true pathologist usually rely on the existence of only a few cancerous sites to rate the whole slide as cancer. Following a similar rule, weakly supervised learning algorithms may be more suited for our case 40-43. One of such algorithms is the multi-instance learning (MIL), which treats the whole slide image (WSI) of each patient as a single label, while the small patches/instances in the WSI are not labelled. This is distinctly different from supervised learnings where each patch is considered as individual label 44-47. Moreover, the interpretability and visualization of the deep-learning networks are essential for clinical applications, e.g., annotated histological results incorporating heatmap and attention mechanism would assist pathologists to locate important regions of interest (ROI) 48-52. Classic semantic segmentation algorithms require precise labeling at the pixel level, which is challenging in most cases, especially for SRS imaging on fresh tissues without corresponding H&E slides to provide pixel-level labeling. Although there have been a number of algorithms that provide deep network interpretability 53-57, they mostly rely on the outputs of specific neurons with non-intuitive visualizations. In contrast, visualization algorithm based on weakly supervised learning can achieve cost-effective pixel-level morphological localization without the need of pixel-level labels. For instance, gradient-weighted class activation mapping (Grad-CAM) is able to produce heatmaps to highlight ROIs related to the corresponding classes 55, 56.

In this study, we designed a 3-step end-to-end SRS image diagnosis pipeline which includes: (1) SRS image acquisition on breast core-needle biopsy tissues; (2) MIL based image classification and diagnosis, by slicing the training dataset into small patches/tiles to train the deep neural network; (3) Image visualization based on Grad-CAM algorithm. Our results proved that SRS microscopy is able to capture the main diagnostic features in frozen section and fresh biopsy tissue of breast cancer. The weakly-supervised MIL model based on 61 biopsy tissues could reach diagnostic accuracy of 95%, higher than conventional supervised learning model. Furthermore, Grad-CAM based visualization and segmentation was realized to locate benign and malignant tissue areas.

## Methods

### Sample preparation

All patients recruited in this study underwent ultrasound guided 14-gauge CNB or vacuum-assisted stereotactic CNB procedures at Fudan University Shanghai Cancer Center (FUSCC). Informed written consent was approved by FUSCC Ethics Committee (No. 050432-4-1911D). Multiple biopsy tissues from the same patient were obtained, one of which was delivered to the microscopy lab for label-free SRS imaging, and the rest specimens were sent for H&E histopathology, which served as the ground truth for the diagnostic result. For SRS imaging, 61 biopsy samples from 59 patients with diagnostic significance were collected, including 26 benign patients and 33 malignant patients, most of them provided one CNB sample each, except for two malignant patients (M19 and M20) who provided two biopsy samples each (Table S1). Fresh CNB tissues were sealed between two coverslips and a perforated glass slide (~0.7 mm thickness, ~ 8 mm diameter hole) for direct SRS imaging. For imaging frozen sections, thin sections of ~20 µm thicknesses were used for SRS imaging without further processing, and adjacent ~5 µm thick sections were sent for H&E staining.

### SRS microscopy

**Figure 1:**
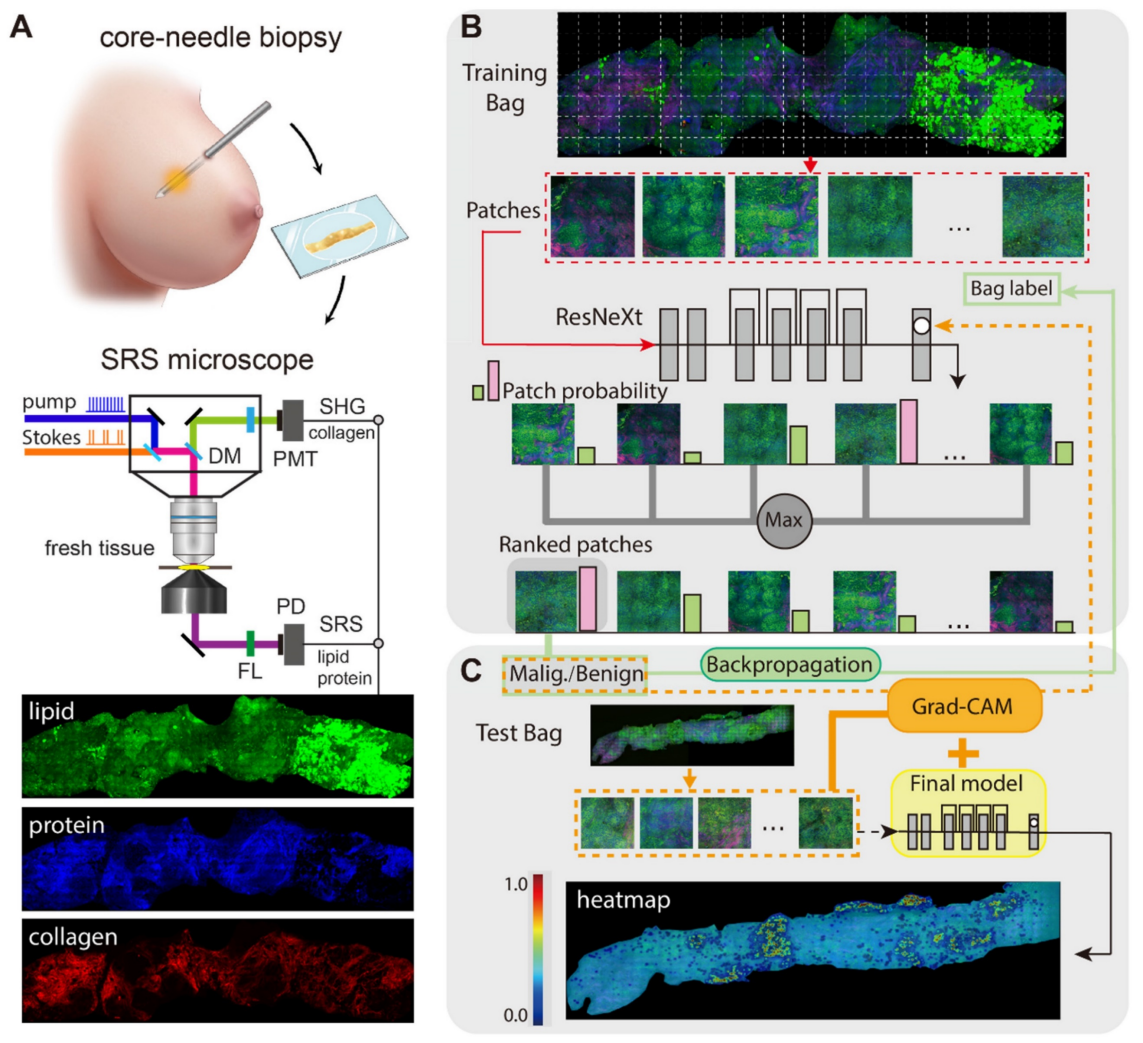
** Schematics of the experimental design.** (A) Imaging breast core-needle biopsy with SRS microscopy, generating distributions of lipid, protein and collagen fibers. DM: dichroic mirror; PD: photodiode; PMT: photomultiplier tube; FL: optical filter. (B) Implementation of the multi-instance learning (MIL) algorithm. The whole-slide SRS image as the training bag was sliced into small patches, and sent into ResNeXt model to provide the ranking result based on the output probability (pink and green bars). Max pooling selected the highest score as the bag's output probability to back-propagation for model optimization (green line). (C) Implementation of Grad-CAM algorithm for heatmap visualization.

Figure 1 illustrates the experimental design of the work. The optical apparatus of our home-built nonlinear optical microscope is shown in Figure 1A and previous publications 26, 30. The dual laser outputs are provided by a commercial femtosecond optical parametric oscillator (OPO, Insight DS+, Newport, CA). The Stokes beam is the fundamental 1040 nm (~150 fs), and the pump beam is the tunable OPO output (680-1300 nm, ~ 120 fs). Both beams are chirped to ~2 ps by passing through long glass rods. The Stokes beam is modulated at the frequency of 20 MHz via an electro-optical modulator (EOM). A motorized delay stage is used to change the Raman frequency using the “spectral focusing” technique. The combined pump and Stokes beam are focused onto the sample through a water immersion objective (UPLSAPO 60XWIR, NA 1.2 water, Olympus) of the laser scanning microscope (FV1200, Olympus). The generated SRS signal is detected by a homemade back-biased photodiode (PD) and demodulated by a lock-in amplifier (LIA) (HF2LI, Zurich Instruments). Second harmonic generation (SHG) signal from collagen is collected by the same objective and detected by a photomultiplier tube (PMT) with a bandpass filter at 520/10 nm. Laser powers at the samples are kept around 30 mW and 40 mW for the pump and Stokes beam, respectively. The pixel dwell time is ~2 μs, and the time to digitize an high-resolution (~350 nm lateral resolution) image of a field of view (FOV, ~180 × 180 μm, 512 × 512 pixels) is ~1 s with multi-channel (2845 cm^-1^ and 2930 cm^-1^ for SRS, plus one SHG channel) parallel detection 26.

### Data preprocessing

The Raman shifts at 2845 cm^-1^ and 2930 cm^-1^ were applied to obtained the lipid and protein SRS images by linear decomposition algorithm based on their spectral differences at the two wavenumbers 23, 25. A composite image was generated by merging the three-channel images: protein channel in blue; lipid channel in green; collagen channel of SHG signal in red. Stitching and tiling was employed to generate the SRS image of the whole breast biopsy-WSI, which usually took ~10-20 min to finish the whole slide. All instances were generated by splitting the WSI with a grid size of 450× 450 pixels. These sliced tiles were used as the input patches for training our model. All patches from WSI were retained, including the tissue areas and empty spaces. No additional data processing algorithm was involved in preparing the dataset.

### MIL based CNN model

Our weakly supervised classification method based on deep CNN model was trained under MIL assumption 46, 58-59, and the training process was illustrated in Figure 1B. ResNeXt50(32X4d) was used as the deep CNN model to provide the inference probability 60, 61. Max-pooling function was used to aggregate the scores of all the instances as a standard MIL pooling layer. Each WSI from our dataset can be recognized as a bag consisting of a number of instances. The malignant WSIs were marked as positive bags, and benign cases were marked as negative bags. Positive bags must process at least one positive instance which was predicted by the CNN classifier, while all the instances in negative bags must be classified as negative. Our model gave a high probability score close to 1 to the top-ranked instance in positive bag, and a low probability score close to 0 to the top-ranked instance in negative bag. A single instance selected from each patient would not be enough to train the model effectively, thus top 15 instances instead of top 1 instance were chosen as the max pooling result in our study.

The MIL-CNN model aims to predict the input WSI as 'negative' or 'positive'. In the training process, the patches were randomly cropped, rotated, and mirrored to increase the size of training dataset. Color normalization for each instance was also performed. In the test step, all the instances of each WSI were fed directly into the trained model after color normalization. In order to reduce the effect caused by unbalanced ratio of positive and negative data size, focal loss function was utilized. Adam optimizer was used to optimize the objective function (loss function), giving specific parameters such as the weight decay, learning rate and exponential decay rates as shown in Table S2. The batch sizes of both training and test sets were set to 32. Epoch step was set to 200.

Due to the limited size of our patient dataset, 5-fold cross validation and three repetitions were applied to train and test our model. The 5-fold dataset was divided into training and test sets at patient level, meaning that the patches from the same patient do not appear in both the training and test sets. Data partition was randomly varied in these three repetitions with different names: 1-split, 2-split and 3-split. Performances of the models were demonstrated by averaging the results of the 5 validation datasets and their corresponding standard errors. In order to compare the performances of different deep-learning models, classification accuracy, precision, recall, F-score and the area under the receiver operating characteristic (ROC) curve were calculated as our model metrics 62. Performances between supervised learning and MIL based weakly supervised learning were compared using the aforementioned metrics.

Training and testing were conducted via the advanced Python-based neural network API, PyTorch, with a TensorFlow backend running on four NVIDIA GeForce 1080 Ti graphical processing units.

### Guided gradient-weight class activation mapping and semantic segmentation

Grad-CAM algorithm functioned following the workflow presented in the Figure 1C, the redder the areas in the heatmap were, the more relevant the area pixels were to the predicted malignancy. Vice versa for the blue color. It is difficult to obtain a complete pathological image heatmap with good visual effect by directly stitching the heatmap results at the patch level. Instead, an averaged heatmap method was developed in our study.

The method to generate the averaged heatmap is shown in Figure S1. Take the WSI size of 1350×1350 pixels as an illustration. The WSI bag is sliced into patches/instances with the patch size of 450 pixels, and shifted with a step size of 150 pixels. Based on these patches, Grad-CAM was employed to obtain the heatmap result of each input patch, and the weight value of each pixel is calculated as the mean value over the times each grid is repeated in the model. The same method was applied for semantic segmentation to calculate the distribution of prediction probability. In order to achieve best average results, the final heatmap results in our work are shown with a step size of 50 pixels.

## Results and Discussion

### SRS reveals histological features in frozen breast tissue sections

**Figure 2:**
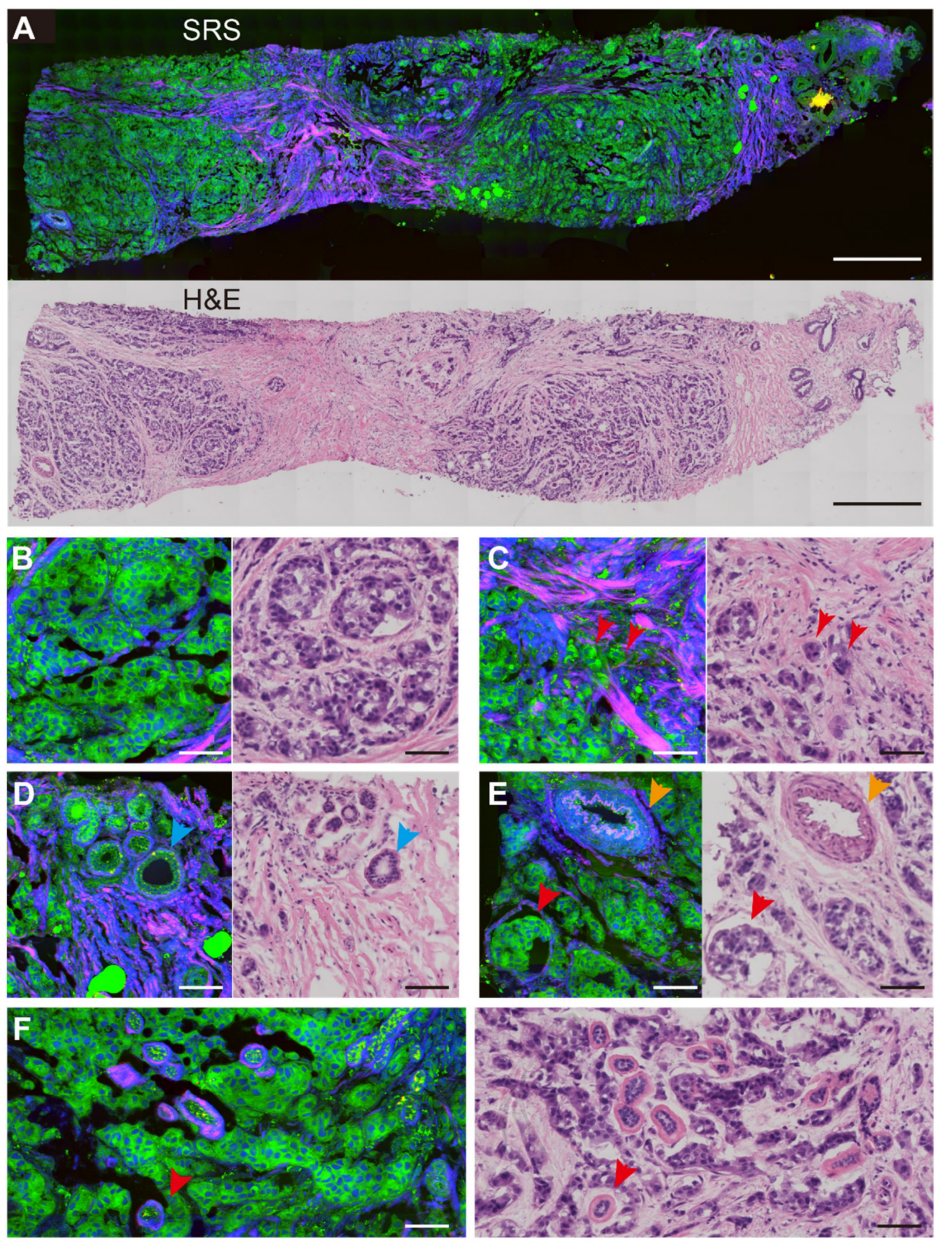
**Comparison of SRS and H&E images in adjacent frozen breast tissue sections.** (A) A whole slide image of a core needle biopsy of invasive ductal carcinoma. Magnified areas, including (B) malignant tumor nests, (C) scattered tumor cells at the tumor boundary (red arrows), (D) normal breast ducts (blue arrows), (E) arterial vessels (yellow arrows) and nearby cancer cells (red arrows), (F) tumor cells infiltrating into the terminal ductal lobular units. SRS color codes: green, lipid; blue, protein; red, collagen fibers. scale bar: (A) 600 µm; (B-F) 60 µm.

Breast CNB specimens were imaged with SRS microscopy to map out the distributions of lipid, protein and collagen fibers. In order to obtain direct comparison between SRS and H&E in revealing important histoarchitectures of breast tissues, we first performed imaging of the two modalities on adjacent thin frozen tissue sections, one of which was imaged with SRS without further processing and the other was sent for H&E staining (Figure 2). Figure 2A shows the typical results of paired SRS and H&E on a biopsy tissue of breast invasive ductal carcinoma. The adjacent sections demonstrate high degrees of consistency at both macroscopic and microscopic levels, showing the capability of SRS of detecting breast tissue histology similar to H&E staining. In particular, the infiltration of tumor cells around the blood vessel and terminal ductal lobular units (TDLU) were clearly characterized. More detailed structures can be visualized in Figure 2B-F through the corresponding magnified SRS and H&E images. Figure 2B-C characterize key histological features of breast cancer, including the cancer nests and individual cancer cells scattered into the surrounding breast tissue (red arrows in Figure 2C). Normal breast histological features can be recognized in Figure 2D-E, such as the normal ducts, arterioles, and TDLU of the breast. As indicated by the blue arrow in Figure 2D, SRS can clearly reveal the double-layered structure of the duct: the glandular epithelium of the inner layer and the myoepithelium of the outer layer. Figure 2E imaged the infiltration of tumor cells around blood vessels (yellow arrow) and tumor clusters (red arrow). The basal membrane and ductal epithelium in the TDLU can also be observed by SRS, as shown by the red arrow in Figure 2F.

### Imaging fresh breast core-needle biopsy with SRS

**Figure 3:**
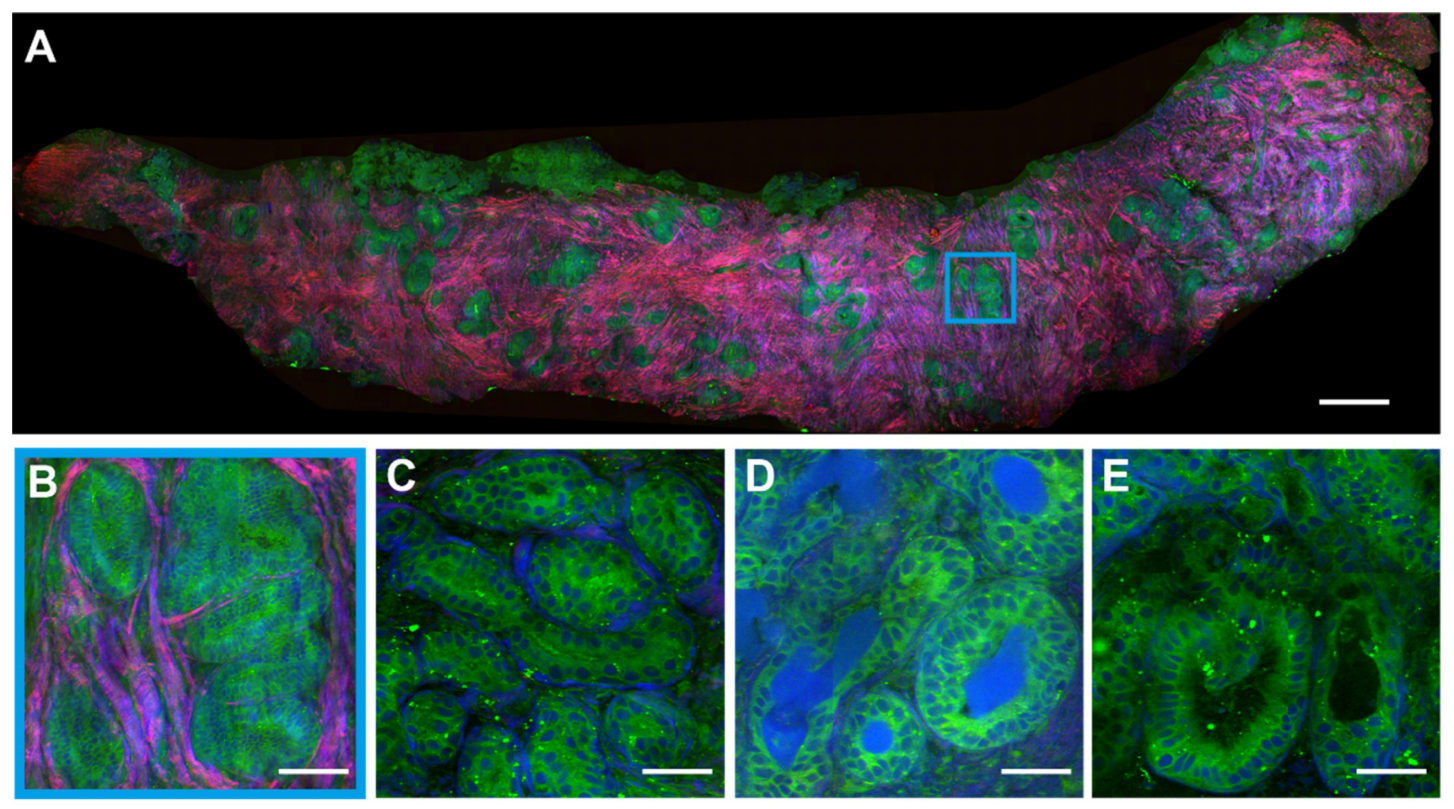
** SRS images of fresh benign breast core needle biopsy**. (A) A whole slide image of fibroadenoma tissue. (B) Breast ductal hyperplasia. (C) Terminal ductal lobule unit. (D) Endoluminal secretions. (E) Open ductal structure of the lumen. Scale bars: (A) 600 µm; (B-E) 60 µm.

**Figure 4:**
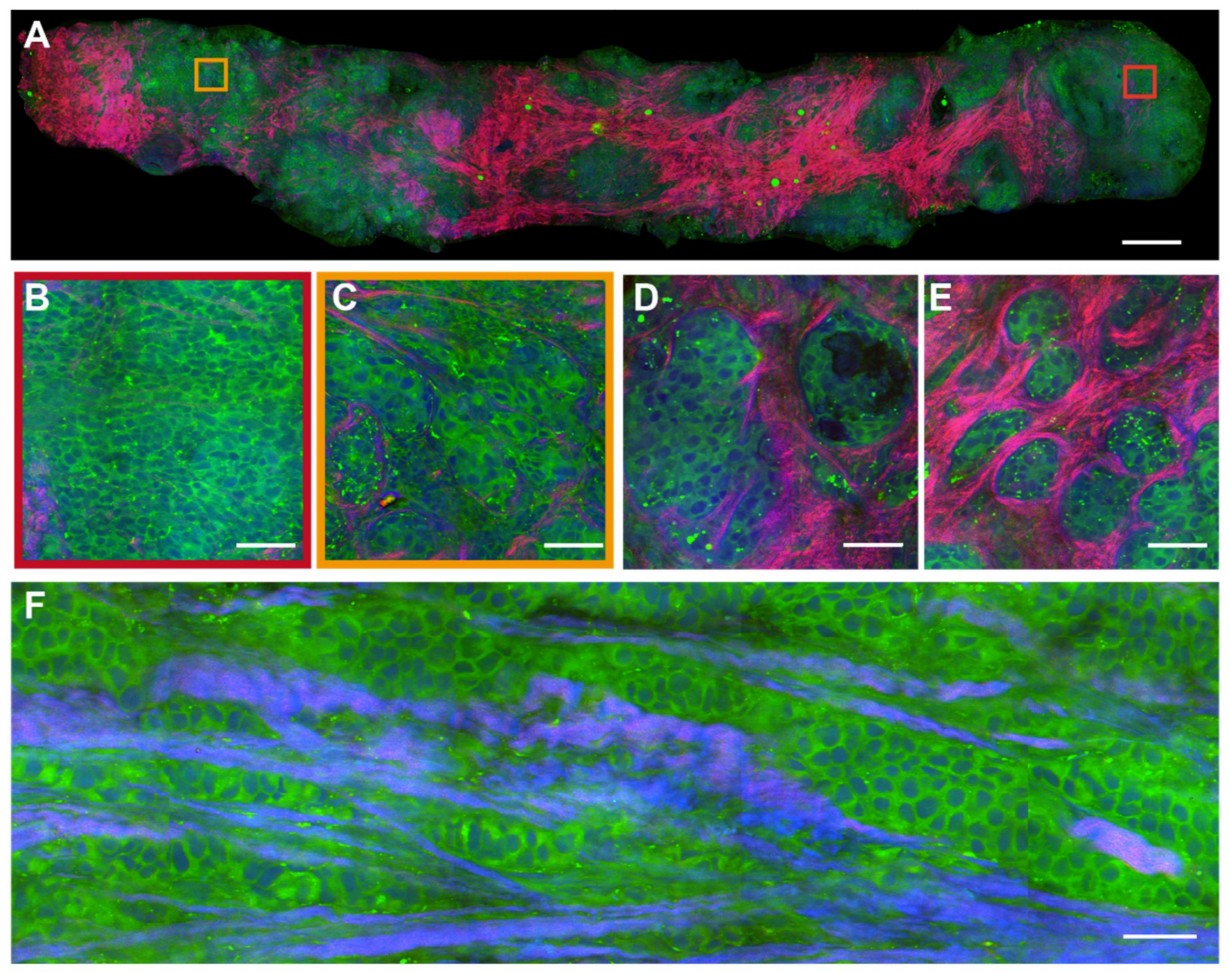
** SRS images of fresh malignant breast core needle biopsy.** (A) A whole slide image of DCIS. (B) Solid pattern tumor cells. (C) Cellular atypia with diverse nucleus sizes. (D) The glandular structure. (E) Infiltration of tumor cells into collagen fibers. (F) Cordlike pattern of carcinoma. Scale bars: (A) 600 µm; (B-F) 60 µm.

We preformed SRS imaging on fresh breast core-needle biopsy to simulate label-free intraoperative histological diagnosis. The key diagnostic features of benign (fibroadenoma) and malignant (ductal carcinoma in situ, DCIS) tissue are well resolved as shown in Figures 3 and 4. In a typical benign CNB specimen (Figure 3A), SRS is able to reveal the overall tissue histoarchitectures with cellular patterns and collagen fiber morphologies. At the microscopic level, it shows ductal epithelial hyperplasia with increased layers, yet the cell morphologies remain normal without the atypia of malignant cancer cells, as shown in the magnified view of the blue square region (Figure 3B). More specific microscopic structures of benign breast tissue can be seen in Figure 3C-E, including the usual breast ductal hyperplasia, TDLU, endoluminal secretions, and open ductal structures of lumen.

In the malignant case (Figure 4A), SRS uncovers the features of cancer cells infiltrating into the surrounding tissues in a cord-like pattern, with obvious cellular atypia. The detailed histological hallmarks of DCIS in the highlighted square areas are presented in Figure 4B-F. The formation of cancer nests with solid-pattern tumor cells (Figure 4B), and cellular atypia including the pleomorphic cell morphology, nuclear size, and micro-clusters (Figure 4C) could be clearly seen. Moreover, various additional tissue histoarchitectures of malignancy could be detected, such as the glandular structure of the carcinoma (Figure 4D), the infiltration of tumor cells into collagen fibers (Figure 4E), and the cord-like patterns of the carcinoma (Figure 4F). These results verified that SRS microscopy was able to identify the intact histological features of fresh unprocessed breast biopsies without potential artifacts introduced by tissue freezing and sectioning processes.

It is worth noting that the breast tissue histology demonstrated large heterogeneous distributions of biochemical components and tissue types. As shown in Figures 2-4, breast tissues feature the existence of connective tissues with abundant collagen fibers and sometimes lipid-rich adipocytes, which do not provide key histological information for cancer diagnosis. And the content ratio of these “non-diagnostic” tissues varies significantly from biopsy to biopsy. As a result, it is more challenging to perform machine-learning based diagnosis for breast cancer, compared to previously studied brain tumors or epithelium-based carcinomas with more homogeneous tissue histoarchitectures 29, 30.

### MIL based classification with improved performance

To find an optimal deep learning model for automated breast cancer classification, we applied weakly supervised learnings to overcome the challenges in supervised learnings. It is less of an issue for supervised learning models to deal with relatively homogeneous tissues as in previous works, where subjective threshold of the classification percentage of the patches within the WSI was required, which may introduce uncertainties of the model performance under different choices of the threshold value 29, 30. Such percentage threshold method may become invalid for highly heterogeneous breast tissues, where only small portion of the cancerous tissue is sufficient to diagnose cancer for the WSI. Contrary to supervised learning, MIL relies on the highest likelihoods of positive instances to determine the overall positive label of the WSI. Hence the logic of MIL algorithm fits better to the diagnostic criteria of breast cancer.

**Figure 5:**
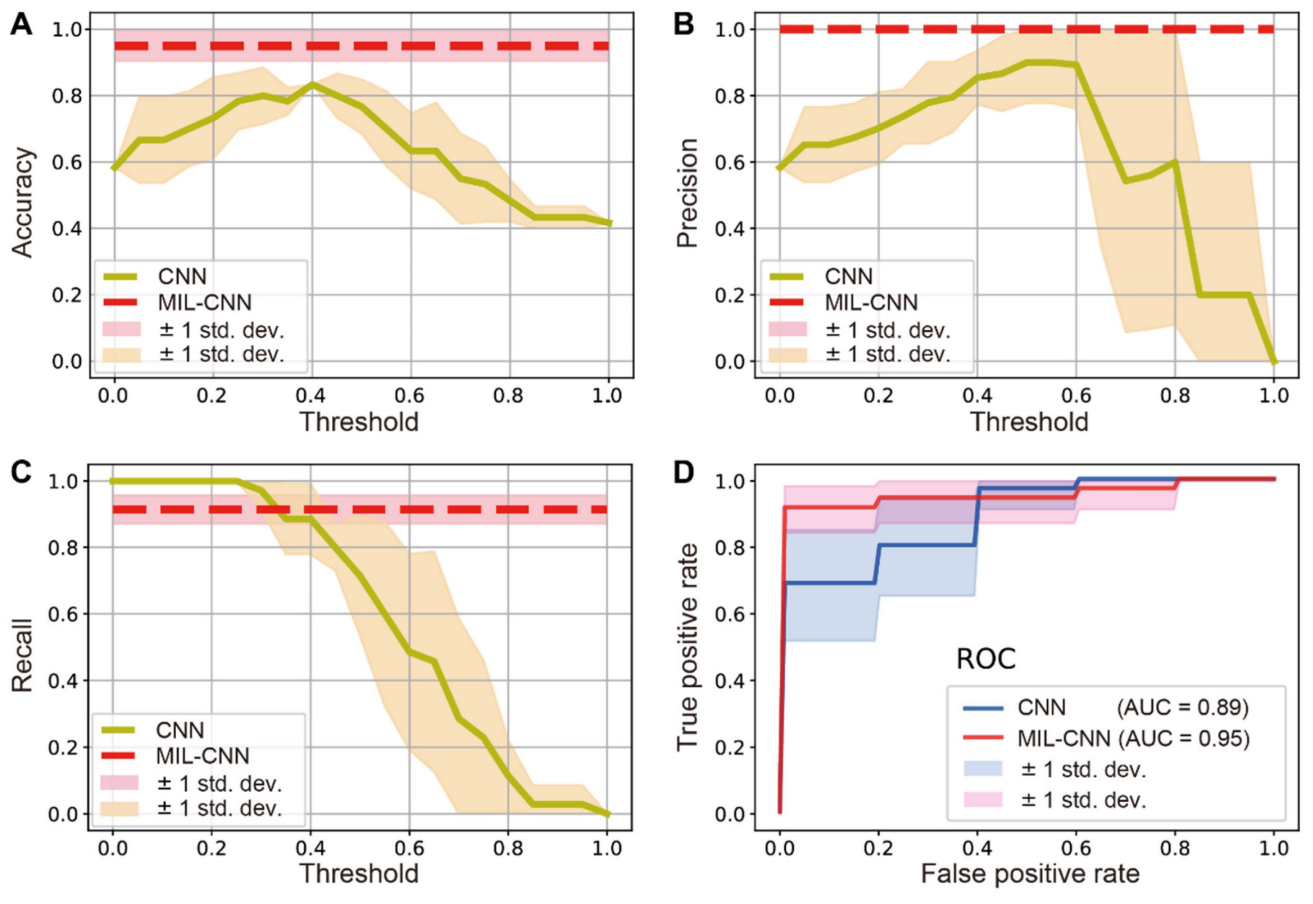
** Comparison between weakly-supervised MIL-CNN and supervised CNN model.** Dependences of the model prediction (A) accuracy, (B) precision and (C) recall on the threshold values. (D) ROC curves of the two models. The shades represent the standard deviation from 5-fold cross validation.

To evaluate the performance of MIL in the classification of breast biopsy, traditional supervised CNN (ResNeXt) and weakly supervised MIL-CNN models were both estimated and compared for the diagnosis of benign/malignancy. The performance of traditional CNN model depends significantly on the threshold values, whereas the performance of MIL-CNN algorithm does not (Figure 5). The results of prediction accuracy and precision indicate that supervised learning achieves the best performances at threshold values between 0.4-0.5, yet could hardly exceed 85% of accuracy and 90% of precision. In contrast, weakly supervised model is able to reach constant high accuracy of ~95% and precision of 100% (Figure 5A-B). The recall curves show that supervised CNN approaches 100% when the threshold is set below 0.3, but it drops rapidly as the threshold value increases. Whereas the recall rate of weakly supervised MIL maintained a stable and high value of ~91% (Figure 5C). Moreover, the ROC curves show that MIL-CNN approaches the point (0,1) faster than supervised CNN with higher aeras under the curve (AUC), indicating its superior performance (Figure 5D), which renders weakly supervised MIL model more reliable in diagnosing breast cancer.

For a more direct comparison, the performances of the two models are summarized and listed in Table 1, with the threshold value set to 0.5. As can be seen, the supervised learning model only performed well in precision (~90%), but all the other results are below 80%. In stark contrast, all three repeats of MIL-CNN model demonstrated consistently high accuracy > 92%, precision > 96%, AUC value > 91%, and recall rate of 91%. The highest evaluation results of MIL were marked bold in Table 1, with the best accuracy of 95%, precision of 100%, recall of 91%, F-score of 95% and AUC of 95%.

**Figure 6:**
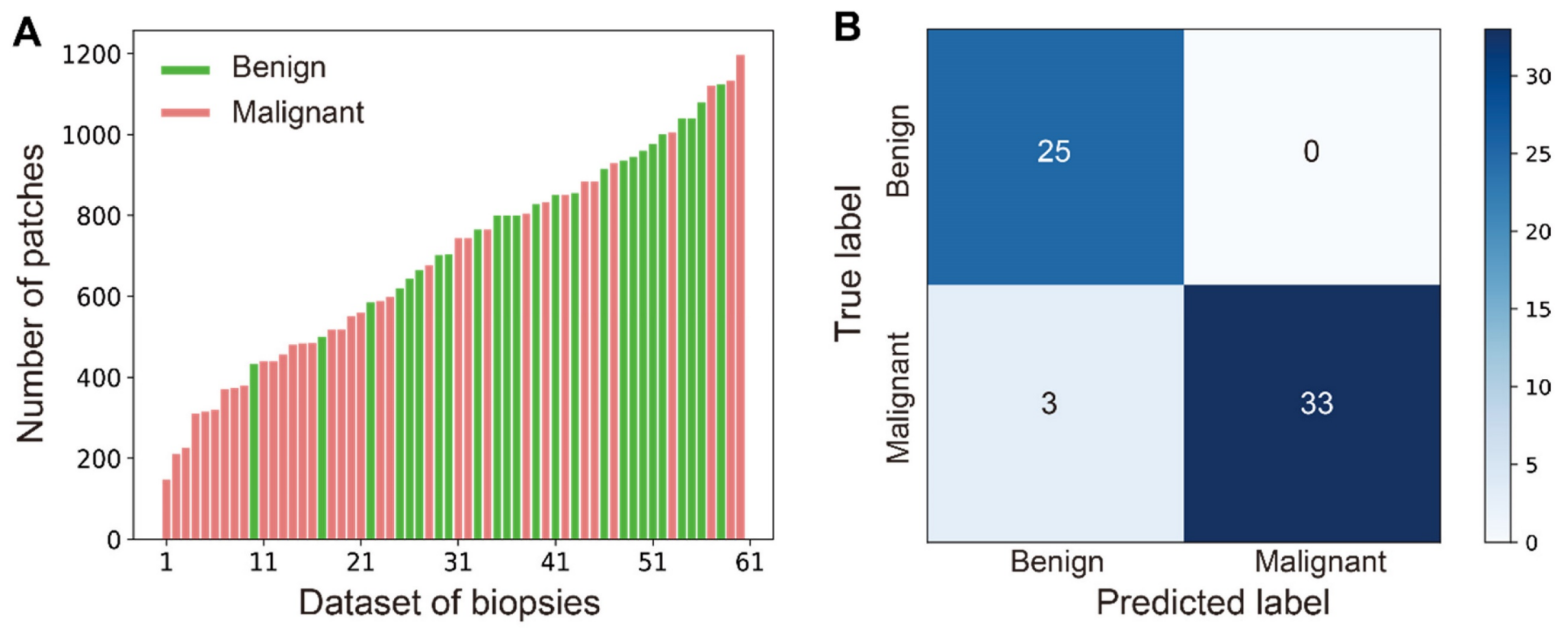
** Robust performance of MIL-CNN.** (A) Wide distribution of the patch number of all the biopsies in the dataset. Green represents benign (negative) and red represents malignant (positive). (B) The confusion matrix of MIL-CNN in 3-split dataset.

In practice, diverse sizes of biopsy tissues and unbalanced number of benign and malignant cases are often encountered, which is also true in our work as shown in the distribution of data size in all studied cases (Figure 6A). The number of patches/instances varied significantly between cases (from 147 to 1197) due to different tissue sizes, and the instance numbers for positive (35) and negative (26) bags are not balanced either. Nonetheless, our MIL-CNN model (3-split) is able to achieve high performances as shown in the above results (Figure 5, Table 1) and the confusion matrix (Figure 6B), indicating that weakly supervised learning is more suited for complex and diverse datasets.

### Heatmap activation and segmentation of SRS histopathology

**Figure 7:**
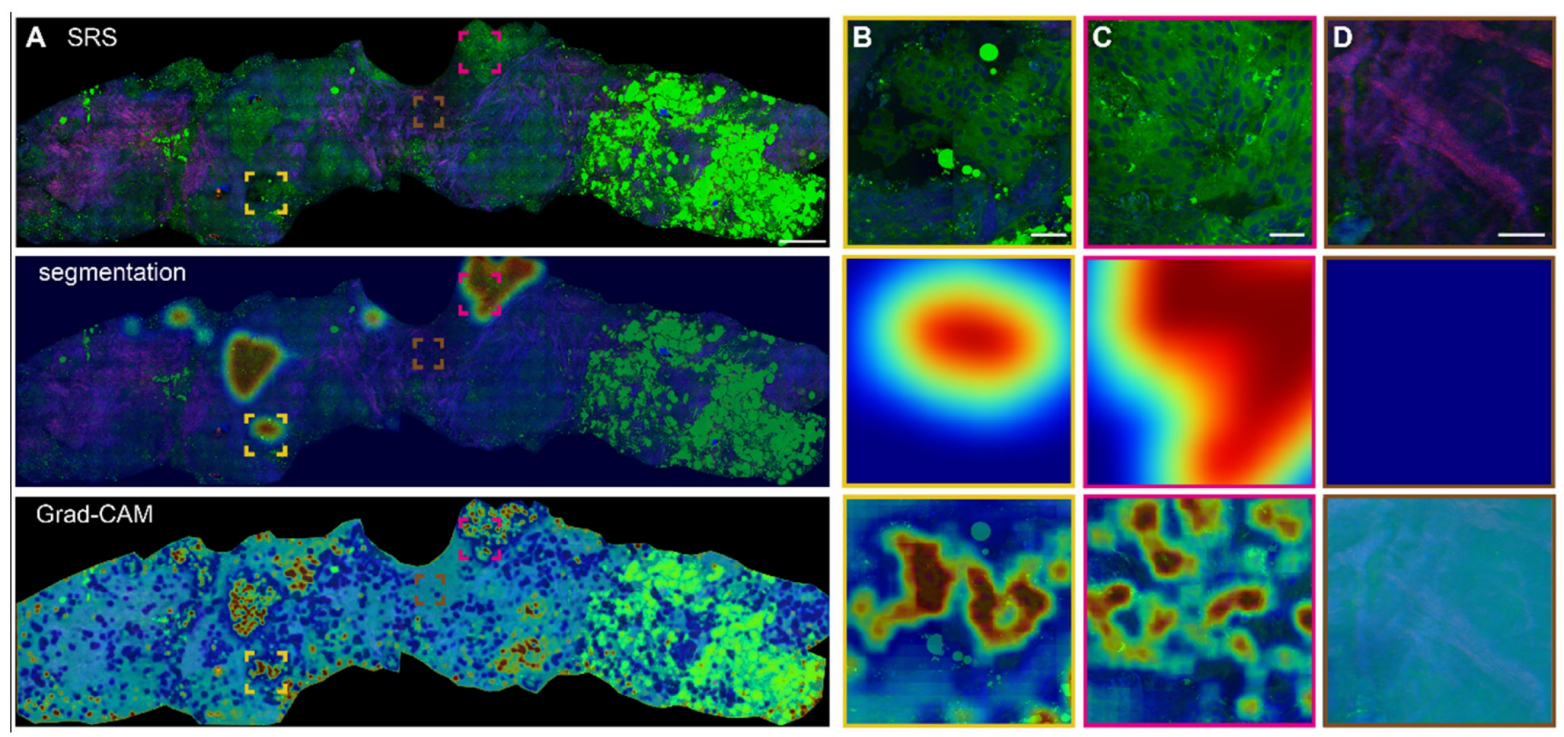
** Heatmap activation and segmentation of breast CNB imaged with SRS.** (A) A whole slide image of a representative breast core needle biopsy, with the overlayed heatmap activation using Grad-CAM and semantic segmentation to identify the cancerous regions. (B-C) Magnified areas with highly activated malignancy. (D) Magnified non-cancer area rich in collagen. The red and blue colors of the heatmap correspond to high and low probabilities of cancer, respectively. Scale bars: (A) 600 µm; (B-D) 60 µm.

Based on the optimized MIL-CNN model for classifying SRS images of breast biopsy, semantic segmentation and Grad-CAM algorithms were further applied to visualize the spatial distribution of diagnostic results generated in the last convolution layer of the neural network. Figure 7A displays the original SRS image (first row) of a fresh breast CNB, along with the heatmaps of highlighted cancerous regions (second row) and Grad-CAM activated regions (third row) superimposed onto the original image. Regions predicted as higher probability of cancer are colored red in the heatmaps, whereas those of lower probability of cancer are colored blue. The heatmaps and the corresponding SRS images are compared to evaluate the classification/activation efficacy. Extracellular matrix composed with abundant collagen fibers, lipid-rich adipocytes and lipid droplets are recognized as less relevant to cancer and appear blue in the heatmaps. Whereas the areas with aggregated cancer cells are labeled as cancerous and appear red. Several representative regions are magnified to show the details in Figure 7B-D. In the identifiable cancerous tissue areas, the model delineates the malignant regions in red (Figure 7B-C), and the heatmap activation provides a fine-grained visualization of the locations that are mostly correlated with cancer. In the areas occupied with dense collagen fibers without cellular structures, the generated heatmap appear cyan, indicating low correlation with cancer (Figure 7D). It is also worth noting that the areas filled with adipocytes (bright green regions at the right side of the biopsy) are recognized as benign. These results confirmed the sensitivities of Grad-CAM and segmentation heatmaps in the spatial identification of different histological classes, and indicated that MIL-CNN model is efficient to resolve cancerous regions to assist diagnosis on breast needle biopsy.

## Discussion and Conclusions

Breast CNB plays an important role in the initial diagnosis and treatment strategy-making of breast cancer. To the best of our knowledge, the current work is the first investigation of SRS microscopy in the histopathological imaging and diagnosis on breast needle biopsy. The capability of SRS in revealing the key features of breast histoarchitectures in both thin frozen tissue sections and unprocessed fresh tissues proves the potential of our method for rapid evaluation of the core-needle specimens, and provide critical information for treatment strategy-making. Moreover, the quick and non-invasive assessment of the biopsy may help reduce the errors in locating the lesions, by rapid screening the correct specimen for further clinical examinations, such as histopathology, immunohistochemistry and genetic tests.

Compared with other label-free tissue histologic imaging methods, such as autofluorescence and optical coherence tomography (OCT) 63, SRS presents better chemical specificity to identify the main biomolecular components of tissues. In addition to the lipid/protein contents, other biochemical components in breast tissues are also relevant to disease states and may be considered for further explorations. For instance, collagen fibers, microcalcifications and carotene contents are found to correlate with breast cancer 14, 62, 64. Compared with the large number of breast cancer studies using spontaneous Raman spectroscopy, SRS has major advantages of rapid and high-resolution imaging of tissue morphology with decent chemical specificity. However, the tradeoff of these advantages is the sacrifice of spectroscopic information. Spontaneous Raman spectroscopy, on the other hand, provides much more spectral details for chemical analysis, but is difficult to obtain spatial information. As expected, the combination of spectral/chemical and spatial/morphological information would help improve the diagnostic power of diseases 62, which could be accomplished by hyper-spectral SRS imaging at the cost of more data acquisition time 65. Therefore, with the current SRS technology, the preferred method for histological imaging is to include lipid and protein to offer just sufficient level of diagnostic information similar to H&E, but with optimized speed for timely diagnosis.

The reasons that supervised learning has worse performances for breast tissue diagnosis are worth discussing. On the one hand, homogeneous labelling on the whole tissue level would inevitably introduce large errors for the patches/tiles that are weakly correlated with cancer (such as collagens and adipocytes). Such labelling errors would degrade prediction accuracy at the patch level. On the other hand, whole-tissue level classification is usually judged by the percentage of patches (with a threshold) that are predicted as benign/malignant, which not only has uncertain dependence on the choice of the threshold, but also suffers from the variation of tissue heterogeneity with varying ratios of collagen areas, as can be seen in Figures 2-3. In order to reduce labelling error, individual patches should be correctly labeled, which would require enormous amount of labeling work for pathologists. Weakly supervised learning avoids the labeling of patches, aiming at high prediction accuracy at the whole-tissue level, even though the prediction accuracy at the patch level might not be high. For future works, semi-supervised learning could be evaluated for breast tissue, which requires the labelling of a small subset of patches with high accuracy, and might reach decent prediction accuracy at both patch and whole-tissue levels 66.

We believe our pursuit of developing an efficient and intelligent diagnostic platform for breast biopsy based on SRS microscopy could be improved in a few ways. Firstly, the current work was only focused on the binary classification of benign and malignancy, it should be extended to more tumor subtypes (such as DCIS) given larger datasets. Secondly, more optimized deep-learning algorithms should be investigated, including new MIL pooling functions 59 and semi-supervised learning 66, to improve the accuracy of classification and segmentation for various tumor subtypes. Moreover, different types of breast tissues other than CNB should also be studied, such as surgical specimens for intraoperative evaluation of resection margins. To fulfill the practical goal of clinical translation, the platform should be more integrated and portable with the development of fiber-based lasers.

In short, we have demonstrated the capability of SRS microscopy in providing high quality histological images of breast CNB, revealing key diagnostic features of breast cancer. Weakly supervised learning using the MIL algorithm has shown much improved diagnostic performance compared with supervised learning models. Furthermore, Grad-CAM based heatmap activation and semantic segmentation has enabled visualization of diagnosis with high spatial resolution. Our work paves the way for deep-learning assisted SRS histopathology of breast cancer, in the potential applications for rapid assessment of needle biopsy and intraoperative diagnosis.

## Supplementary Material

Table S1, clinical information of patients and biopsies; Table S2, details of the MIL-CNN model optimization procedure; Figure S1, the averaging method for generating intact heatmap of SRS image.

## Figures and Tables

**Table 1:** ** Comparison between supervised CNN and weakly-supervised MIL-CNN model.** The model performances at patient-level were evaluated on the 3-split dataset for comparison. Bold numbers were the highest scores for MIL-CNN model. All numbers are the mean values and standard deviations of the test dataset under 5-fold cross-validation.

Method	Accuracy	Precision	Recall	F-score	AUC
MIL-1-split	**0.95±0.05**	**1.00±0.00**	**0.91±0.08**	**0.95±0.04**	**0.95±0.05**
MIL-2-split	0.93±0.05	0.97±0.03	**0.91±0.08**	0.94±0.05	0.92±0.06
MIL-3-split	**0.95±0.05**	**1.00±0.00**	**0.91±0.08**	**0.95±0.04**	**0.95±0.05**
CNN-3-split	0.77±0.09	0.90±0.09	0.71±0.13	0.77±0.11	0.79±0.08

## Abbreviations

CNB: core-needle biopsy
SRS: stimulated Raman scattering
SHG: second-harmonic generation
H&E: hematoxylin and eosin
MIL: multi-instance learning
CNN: convolutional neural networks
Grad-CAM: gradient-weighted class activation mapping
DCIS: ductal carcinoma in situ
TDLU: terminal duct-lobular units
WSI: whole slide image
ROI: regions of interest
ROC: receiver operating characteristic
AUC: area under the curve
