# Supplementary material for: Histological diagnosis of unprocessed breast core-needle biopsy via stimulated Raman scattering microscopy and multi-instance learning
Source: Theranostics. 2023 Feb 21;13(4):1342–54. doi: 10.7150/thno.81784 (PMC10008736; doi:10.7150/thno.81784)
Supplement: Supplementary file 1 — Table S1, clinical information of patients and biopsies; Table S2, details of the MIL-CNN model optimization procedure; Figure S1, the averaging method for generating intact heatmap of SRS image. [file thnov13p1342s1.pdf]

# Supplementary Material

**Table S1.** Patients' information.

| Malignant (total 33 patients, 35 biopsies) | | Benign (total 26 patients, 26 biopsies) | |
|---|---|---|---|
| **Number** | **Diagnosis** | **Number** | **Diagnosis** |
| M01 | IDC | B01 | FADS |
| M02 | IDC | B02 | FAD |
| M03 | DCIS | B03 | FAD |
| M04 | IDC | B04 | FAD |
| M05 | IDC | B05 | FAD |
| M06 | IDC | B06 | FADS |
| M07 | IDC | B07 | FADS |
| M08 | IDC | B08 | FADS |
| M09 | IDC | B09 | FADS |
| M10 | IDC | B10 | FADS |
| M11 | IDC | B11 | FAD |
| M12 | DCIS | B12 | FADS |
| M13 | IDC | B13 | FAD |
| M14 | IDC | B14 | FADS |
| M15 | IDC | B15 | FAD |
| M16 | IDC | B16 | FAD |
| M17 | IDC | B17 | FAD |
| M18 | IDC | B18 | FAD |
| M19 | IDC (2 biopsies) | B19 | FAD |
| M20 | IDC (2 biopsies) | B20 | FAD |
| M21 | IDC | B21 | SA |
| M22 | IDC | B22 | FAD |
| M23 | DCIS | B23 | FAD |
| M24 | IDC | B24 | FAD |
| M25 | IDC | B25 | FAD |

| | | | |
|---|---|---|---|
| M26 | IDC | B26 | FAD |
| M27 | IDC | | |
| M28 | IDC | | |
| M29 | IDC | | |
| M30 | IDC | | |
| M31 | IDC | | |
| M32 | IDC | | |
| M33 | IDC | | |

Abbreviations:

IDC: invasive ductal carcinoma

DCIS: ductal carcinoma in situ

FAD: fibroadenoma

SA: breast sclerosing adenosis

FADS: fibroadenosis

**Table S2.** Details for the MIL-CNN model optimization procedure.

| **Class** | **Value** |
|---|---|
| Criteria | Focal loss |
| Optimizer | Adam |
| $\beta_1$ | 0.9 |
| $\beta_2$ | 0.999 |
| Learning rate | 0.001 |
| Weight decay | 0.0001 |
| Epochs | 200 |
| Batch size | 32 |
| Number of top tiles | 15 |

**Figure S1.** The averaging method for generating intact heatmap of SRS image. WSI size here is 1350×1350 pixels, patch size is 450×450 pixels, step size is 150 pixels.

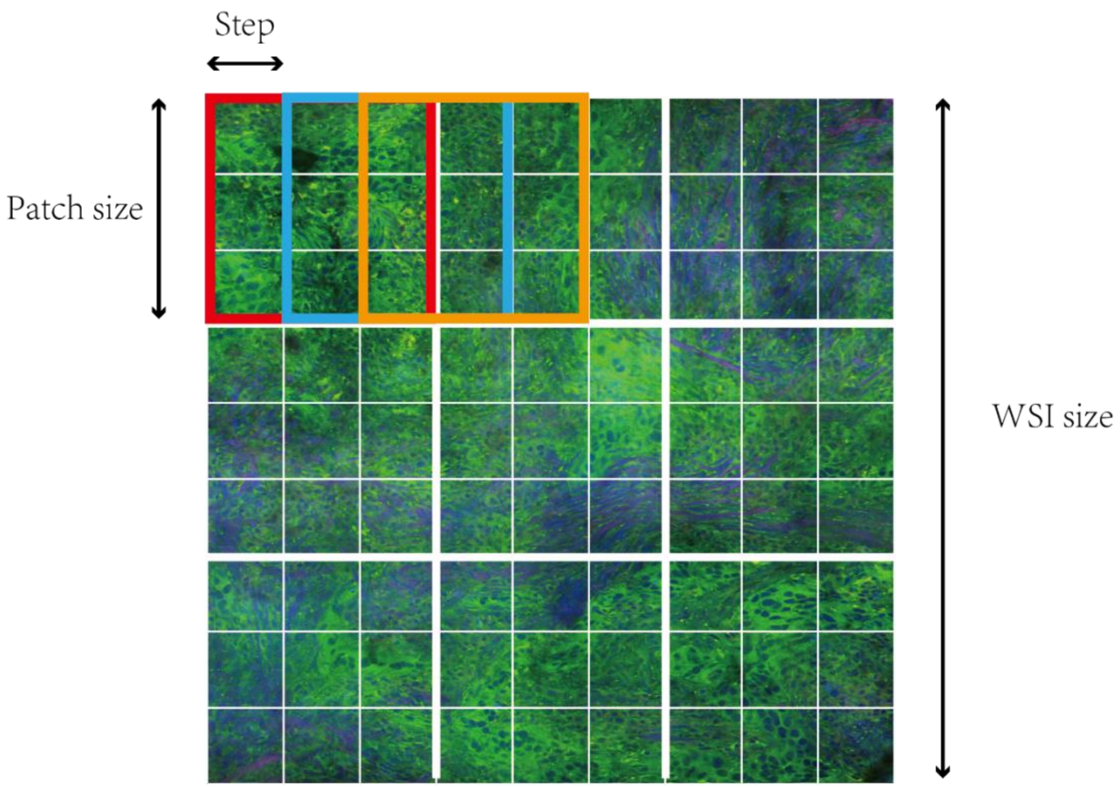


| | | | | | | | | |
|---|---|---|---|---|---|---|---|---|
| 1 | 2 | 3 | 3 | 3 | 3 | 3 | 2 | 1 |
| 2 | 4 | 6 | 6 | 6 | 6 | 6 | 4 | 2 |
| 3 | 6 | 9 | 9 | 9 | 9 | 9 | 6 | 3 |
| 3 | 6 | 9 | 9 | 9 | 9 | 9 | 6 | 3 |
| 3 | 6 | 9 | 9 | 9 | 9 | 9 | 6 | 3 |
| 3 | 6 | 9 | 9 | 9 | 9 | 9 | 6 | 3 |
| 3 | 6 | 9 | 9 | 9 | 9 | 9 | 6 | 3 |
| 2 | 4 | 6 | 6 | 6 | 6 | 6 | 4 | 2 |
| 1 | 2 | 3 | 3 | 3 | 3 | 3 | 2 | 1 |
